# Supplementary material for: A deep proteomics perspective on CRM1-mediated nuclear export and nucleocytoplasmic partitioning
Source: eLife. 2015 Dec 17;4:e11466. doi: 10.7554/eLife.11466 (PMC4764573; doi:10.7554/eLife.11466)
Supplement: Supplementary file 5. — DOI: http://dx.doi.org/10.7554/eLife.11466.018 [file elife-11466-supp5.docx]

| **Previous**  **Categories** | **BioGrid**  **(163)** | **NESdb**  **Human**  **(135)** | **NESdb**  **In doubt**  **(16)** | **Thakar, Karaca *et al.* 2013*,***  **(143)** | **Combined**  **Previous** | **Not in any database** |
| --- | --- | --- | --- | --- | --- | --- |
| **Cargo A (543)** | 14 | 23 | 1 | 30 | 57 | 486 |
| **Cargo B (288)** | 7 | 12 | 2 | 17 | 31 | 247 |
| **Cargo C-low ab. (239)** | 1 | 6 | 1 | 6 | 12 | 227 |
| **Ambiguous (2419)** | 51 | 22 | 2 | 54 | 125 | 2294 |
| **Non-binder (880)** | 19 | 11 | 2 | 4 | 30 | 850 |
| **NTRs (19)** | 2 | 0 | 0 | 8 | 10 | 9 |
| **NUPs (20)** | 5 | 0 | 0 | 3 | 7 | 13 |
| **Not Found** | 60 | 60 | 7 | 19 |  | |

***Table S5*** compares newly identified CRM1 cargoes from human HeLa cells with previous identifications listed in BioGrid (Stark *et al.*, 2006; Chatr-Aryamontri *et al.*, 2015), the NESdb database (Xu *et al.*, 2012a, 2012b), and reported by (Thakar *et al.*, 2013). Note that there is overlap between these previous entries and that 960 (90.6%) of the 1060 "category A-C" cargoes are new assignments. The fraction of new assignments for yeast and *Xenopus* cargoes is even higher (95% and 99.5 %, respectively).

A repeatedly noticed problem is that numerous published NESs and cargo assignments turned out to be incorrect, mostly because the assumed NESs are buried in the hydrophobic core (see Stüven *et al.*, 2003 (Hantschel *et al.*, 2005; Xu *et al.*, 2012b) and discussion therein). Our data can therefore also be seen as an unbiased validation of previous assignments. We could clearly confirm 100 of these, but failed to detect any CRM1•cargo-like interaction for another 30. We regard Nups and other NTRs (like importin α or transportin) not as CRM1-cargoes.
